# Malignant Struma Ovarii: Good Response after Thyroidectomy and ^131^I Ablation Therapy

**DOI:** 10.4137/cmo.s410

**Published:** 2008-02-29

**Authors:** Erica W.M. Janszen, Helena C. van Doorn, Patricia C. Ewing, Ronald R. de Krijger, Johannes H.W. de Wilt, Boen L.R. Kam, Wouter W. de Herder

**Affiliations:** 1 Department of Obstetrics and Gynecology, sector of gynecologic oncology, Erasmus Medical Center, P.O. Box 2040, 3000 CA, Rotterdam, The Netherlands; 2 Department of Pathology, Erasmus Medical Center, Rotterdam, The Netherlands; 3 Department of Surgery, sector of surgical oncology, Erasmus Medical Center, Daniel den Hoed Clinic, Rotterdam; 4 Department of Nuclear Medicine, Erasmus Medical Center, Rotterdam; 5 Department of Internal Medicine, Sector of endocrinology, Erasmus Medical Center, Rotterdam, The Netherlands

**Keywords:** malignant struma ovarii, radioiodine therapy, thyroidectomy, germ cell tumors, multidisciplinary approach

## Abstract

**Background:**

Malignant struma ovarii is a rare malignant germ cell tumor of the ovary. Due to the rarity of this disease, treatment has not been uniform throughout the published literature.

**Cases:**

We present three cases of malignant struma ovarii. Following primary surgery, all were subsequently treated with thyroidectomy and ^131^I ablation therapy, two patients as first line management, one following the occurrence of metastatic disease.

**Conclusion:**

Histological diagnosis of malignant struma ovarii is similar to that of well differentiated thyroid carcinoma (WDTC). In line with the latest advice on treatment of WDTC, we believe that the best option for patients with malignant struma ovarii is surgical removal of the ovarian lesion followed by total thyroidectomy which allows the exclusion of primary thyroid carcinoma, and in addition, allows radioiodine (^131^I) ablation therapy for (micro) metastasis. After thyroidectomy, thyroglobulin can be used as a tumor marker for follow-up. Moreover, nuclear medicine imaging using radioiodine (^123^I) can be performed to demonstrate metastatic carcinoma. A multidisciplinary approach is essential.

## Introduction

The diagnosis of malignant struma ovarii is based on classical criteria for papillary or follicular thyroid carcinoma including overlapping ground glass nuclei in the former and vascular space invasion and capsular penetration in the latter. Malignancy is more likely in tumors larger than 16 cm or when metastatic seeding of the peritoneum is present, so called “strumosis” (Young, 1993; Ayhan et al. 1993). The peak incidence lies between 40 and 60 years of age (Ross DS. 2005 UpToDate^®^ ‘Struma ovarii’ http/www.utdol.com/utd/content/). Patients often present with pelvic pain or irregular menstrual bleedings (Devaney et al. 1993; DeSimone et al. 2003; Makani et al. 2004), whereas a minority (10%) has symptoms consistent with hyperthyroidism. Transvaginal examination may reveal a cyst, often with septae or solid areas. This may mimic an endometrial- or dermoid cyst.

The rarity of tumors such as malignant struma ovarii precludes any uniform management suggestions, other than attempting complete cytoreductive surgery. Surgery is needed for diagnosis, surgical staging, and definitive treatment. Therapy depends on age: for women of childbearing age it is recommended to preserve uterus and contra lateral ovary if they appear to be normal. Total abdominal hysterectomy and bilateral salpingo-oophorectomy should be performed in women who have completed childbearing. Adjuvant therapies should be geared toward the transformed component.

Metastases from malignant struma ovarii are rare (5%) (Willemse et al. 1987; Rose et al. 1998; DeSimone et al. 2003). Metastases from a primary thyroid carcinoma simulating struma ovarii are even rarer (Makani et al. 2004).

This article will review three patients with malignant struma ovarii, its therapy and follow-up, who were treated in our clinic. In addition recommendations regarding potential treatment options are given.

## Case Reports

### Patient A

A 33-year-old, para 1 complained of progressive lower abdominal pain and stress incontinence. Physical examination revealed an immobile, firm tumor extending in the right lower quadrant of the abdomen to 2 cm below the umbilicus. Transvaginal ultrasound revealed a 10 × 15 cm cyst with solid parts. A laparotomy was performed; the right adnex, adherent to the right pelvic wall, was removed. Frozen section of this partly cystic, partly solid mass gave no definite diagnosis, therefore the uterus and left adnex were removed (TAH + BSO). Macroscopically all tumor was removed. Histology showed the follicular variant of a papillary thyroid carcinoma arising in a struma ovarii.

**Figure 1:**
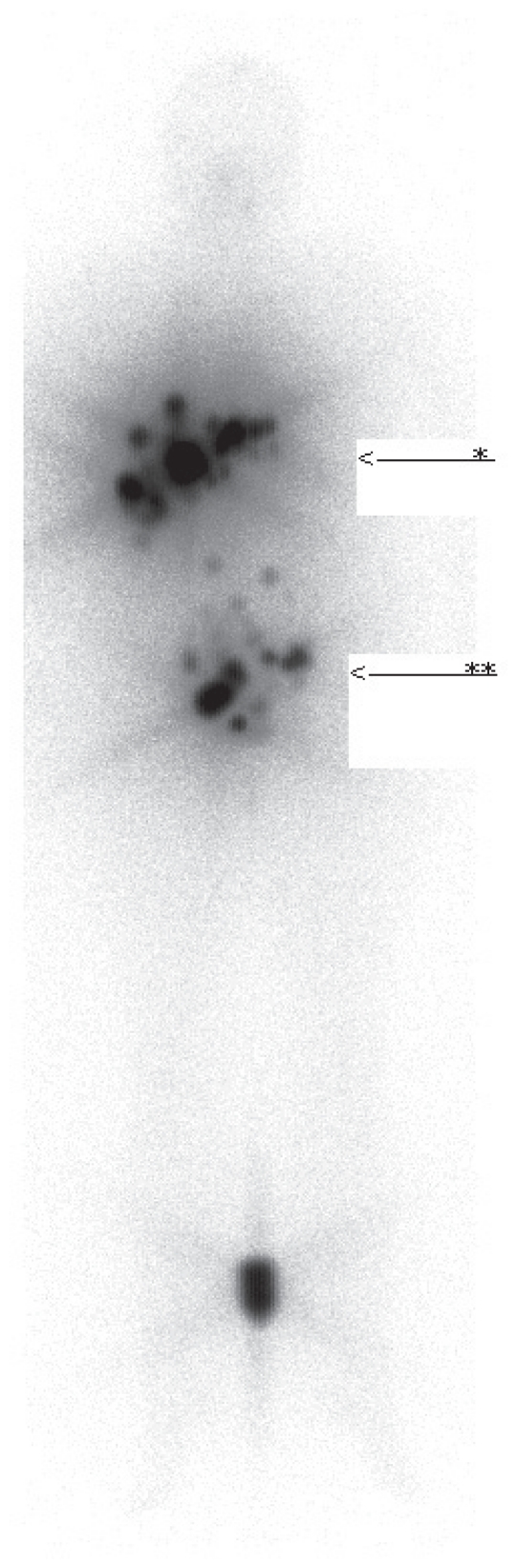
Total body scintigraphy eight days after 5550 MBq. ^131^I treatment. Intense and multifocal laesions in the liver (*) and abdomen (**).

After three years follow-up the patient again complained of abdominal pain and bladder problems. There was no weight loss and no symptoms of anxiety. Both ultrasound and MRI studies showed multiple liver metastases and suspicious lymph nodes along the right iliac artery. Fine needle biopsy confirmed the presence of metastases from a papillary thyroid carcinoma. In order to treat these metastases with radioiodine a total thyroidectomy was performed. Histology of the thyroid gland was normal. Postthyroidectomy thyroglobulin levels were 607 μg/l (normal value 1–20 μg/l). Subsequently she received a total of three doses of ^131^I in doses of 5550 MBq, 1850 MBq and 5550 MBq respectively with intervals of 6 months. Each treatment was followed 24 hours later by a total body scintigram: liver uptake diminished over time from 40% to 17% (see Fig. 1). No new sites of tumor formation occurred over time. With this treatment she reached stable disease, which up to now lasts for four years. Serum thyroglobulin levels have stabilized at levels amounting to 112 ug/l since the last ^131^I therapy.

### Patient B

A 52-year-old, para 3, postmenopausal woman was found to have an ovarian cyst at routine check-up. There was unexplained weight loss of 11 kilograms during the preceding six months. Thyroid stimulating hormone (TSH) serum levels were normal. Ultrasound showed a 10 × 8 cm cyst with partitions and solid areas in the left ovary.

**Figure 2:**
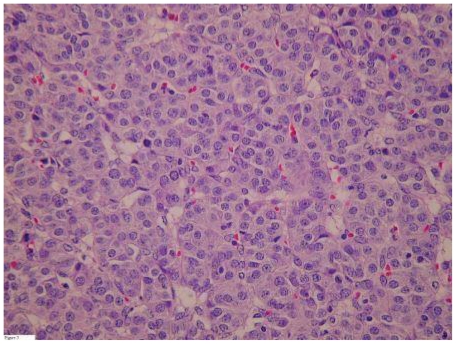
Follicular carcinoma in a struma ovarii.

**Figure 3:**
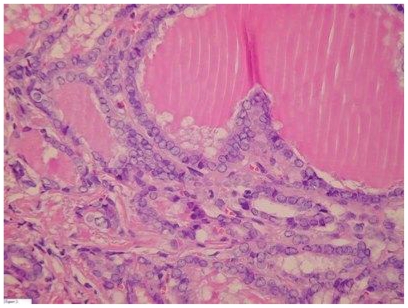
Papillary thyroid carcinoma in thyroid gland.

Laparotomy revealed a large grey, smooth cyst about 15 cm in diameter originating from the left ovary. A small amount of ascites was present. Frozen section of the left ovary was inconclusive, so bilateral salpingo-oophorectomy was performed. Histology revealed follicular carcinoma in a struma ovarii (see Fig. 2). Subsequently a total thyroidectomy was performed, showing a papillary thyroid micro carcinoma of 3 mm (see Fig. 3). ^123^I-scintigraphy showed only little uptake in the thyroid gland. Thyroglobulin serum levels were undetectable. Ablative therapy was given with a dose of 5550 MBq ^131^I. Two years after this treatment there is no evidence for recurrence.

### Patient C

**Figure 4:**
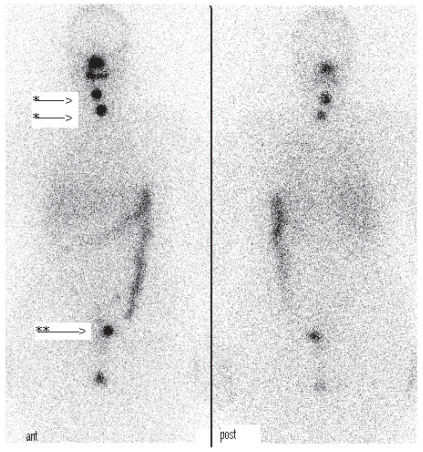
Total body scintigraphy eight days after 5550 MBq. ^131^I treatment. Two small spots in the region of the former thyroid are visible, probably remnant (*). In the pelvic region, there is pathological uptake visible just left of the bladder (**). This could be residual tumor activity.

A 32-year-old, para 0 complained about prolonged diarrhea without weight loss. She had lower abdominal pain. She had regular withdrawal bleedings with oral contraceptives. The patient wished to conceive in the future. Ultrasound revealed ascites and a cystic left ovary 8 × 4 cm. At laparotomy the cyst was found to be fused with the serosa of the small intestines. In order to allow complete excision a colostomy was performed along with a unilateral oophorectomy. Histology revealed a papillary carcinoma in struma ovarii. After total thyroidectomy, ^131^I ablation therapy with 5550 MBq and ^123^I scintigraphy followed. The scintigram showed minimal pathological rest-activity behind the bladder (see Fig. 4). Because the patient had anti-thyroglobulin antibodies, serum thyroglobulin could not be used as a tumor marker. Six months after the first operation the colostomy was discontinued. Inspection of the abdomen did not reveal any macroscopic tumor. Recently a total body scintigram was performed, which did not show evidence for remaining disease. The pathological rest activity behind the bladder might have disappeared due to the lasting effect of ^131^I ablation therapy.

## Discussion

We reviewed three patients, two with papillary thyroid carcinoma, and one with follicular thyroid carcinoma in struma ovarii. Two of them presented with pain, two experienced symptoms (weight loss and diarrhea) which might have been caused by hyperthyroidism although TSH levels at the time were normal. All had enlarged cystic ovaries with an average diameter of 11 cm. One of our patients had a papillary thyroid micro carcinoma in the thyroid gland. In autopsy series such small papillary carcinomas are found in about 25% (Yamamoto et al. 1990). Since histology in the thyroid and ovary differed, the struma ovarii was considered a primary tumor and not a metastasis.

Ultrasound can not specifically identify struma ovarii, however one must consider this diagnosis especially in solid looking teratomas. Pre-operative diagnosis of struma ovarii is only possible in patients with hyperthyroidism by measurement of serum TSH in combination with ^123^I scintigraphy (Masuda et al. 2001). If one suspects struma ovarii, serum levels of free T4, thyroglobulin and TSH should be measured. In none of our patients this was done. Diagnoses were made on histology.

There is no universal therapy for malignant struma ovarii throughout the published literature. Surgery varies from laparoscopic unilateral oophorectomy to TAH + BSO with omentectomy. Malignant struma ovarii shows the same morphological features on histology as well differentiated (follicular and papillary) thyroid carcinoma (WDTC). Like most authors we adhere to the recommendations given by Devaney and co-workers to use the same histological criteria to diagnose malignant struma ovarii as are used for tumors in the thyroid gland (Devaney et al. 1993; DeSimone et al. 2003; Makani et al. 2004). Likewise we share the opinion that therapy and follow up of malignant struma ovarii should be based on criteria for the treatment and follow up of WDTC. A recent consensus report on WDTC by Pacini and co-workers suggests that radioiodine can reduce recurrences and possibly mortality when there is uncertainty about the completeness of thyroid surgery (Pacini et al. 2005). According to this report radioiodine is definitely indicated in patients with distant metastases, incomplete tumor resection, tumors >1 cm, suboptimal surgery (no lymph node resection), young age (<16 years), and/or unfavorable histology. Especially high-risk patients with T3, T4 or T × N1 or T × N × M1 tumors demonstrated significantly reduced recurrence and mortality rates at 20 years after ^131^I treatment as compared to patients who have not undergone ablative radioiodine treatment (Tubiana et al. 1985; Tsang et al. 1998; Pacini et al. 2005). Applying these criteria to malignant struma ovarii would result in thyroidectomy followed by postoperative ^131^I ablation therapy for most patients, since tumor size would exceed 1 cm in most, and lymph node resection is seldom performed. Total thyroidectomy following surgical removal of the ovarian lesion allows the exclusion of primary thyroid carcinoma with metastasis to the ovary and in addition, allows radioiodine (^131^I) therapy for treatment of (micro) metastasis. After thyroidectomy, thyroglobulin can be used as a tumor marker for follow-up. Moreover, nuclear medicine imaging using radioiodine (^123^I) can be performed to demonstrate metastatic thyroid carcinoma. Unfortunately at the time patient A was diagnosed and treated we did not routinely perform thyroidectomy followed by nuclear medicine imaging using radioiodine (^123^I) as we did in patients B and C. This approach might have led to prevention or earlier detection of metastases. This patient shows that after recurrence ^131^I therapy has a beneficial effect on extensive metastatic disease however she is not disease free. Beneficial effects of ^131^I therapy have also been demonstrated by DeSimone and co-workers who reviewed the literature on malignant struma ovarii in a total series of 24 patients (DeSimone et al. 2003). This review reports a recurrence rate after initial complete response of 35%. All recurrences occurred in patients without primary adjuvant therapy. This supports us in the believe that ^131^I therapy should be first line treatment. The above described advantages outweigh the disadvantage of performing a thyroidectomy with its low morbidity (recurrent laryngeal nerve damage (<1%), hypocalcemia (2%) and thyroid replacement therapy). The practitioner should be aware that due to competition between iodine and radioiodine, no iodine (as disinfectant or contrast medium) should be used 6 weeks prior to ^131^I ablation therapy or ^123^I scintigraphy.

Side-effects of ^131^I treatment are usually mild and most are reversible (Pacini et al. 2005). Transient amenorrhea and an early age of menopause have been observed (Ceccarelli et al. 2001). Studies of pregnancy outcome after radioiodine treatment reveal no deleterious effects, although there is an increased risk of miscarriage when conception occurs within 6 months after the last treatment (Schlumberger et al. 1996). Fertile women should be advised about adequate contraception during treatment, and until 6 months thereafter.

## Conclusion

Malignant struma ovarii is a rare gynecological endocrine-oncological disorder. Due to its rarity there is little information about the natural course of this disorder after surgical resection and the best postoperative treatment modalities. If the diagnosis of malignant struma ovarii can be made at the time of surgery, a complete surgical removal of the tumor should be done. In line with the treatment and follow-up of WDTC we believe that the best option for patients with malignant struma ovarii larger than one cm is total thyroidectomy followed by ^131^I ablation therapy. After ^131^I ablation any detectable serum thyroglobulin points to persistent or recurrent disease. After ablation, the highly sensitive post-ablation total body scan can demonstrate completeness of surgical excision or indicate metastatic disease. A multidisciplinary approach is essential, whereby the gynecologist, surgeon, endocrinologist, pathologist and nuclear physician are working closely together.
